# Aurora kinase inhibitors: Progress towards the clinic

**DOI:** 10.1007/s10637-012-9798-6

**Published:** 2012-02-18

**Authors:** Madhu Kollareddy, Daniella Zheleva, Petr Dzubak, Pathik Subhashchandra Brahmkshatriya, Martin Lepsik, Marian Hajduch

**Affiliations:** 1 Laboratory of Experimental Medicine, Institute of Molecular and Translational Medicine, Palacky University, Puskinova 6, Olomouc, 77520 Czech Republic; 2 Cyclacel Ltd, 1 James Lindsay place, Dundee, DD1 5JJ UK; 3 Institute of Organic Chemistry and Biochemistry, v.v.i. and Gilead Sciences and IOCB Research Center, Academay of Sciences of the Czech Republic, 166 10 Prague, Czech Republic

**Keywords:** Aurora kinases, Aurora kinase inhibitors, Cell division, Resistance, Mitosis, Serine/threonine kinases, Spindle pole

## Abstract

The Aurora kinases (serine/threonine kinases) were discovered in 1995 during studies of mutant alleles associated with abnormal spindle pole formation in *Drosophila melanogaster.* They soon became the focus of much attention because of their importance in human biology and association with cancer. Aurora kinases are essential for cell division and are primarily active during mitosis. Following their identification as potential targets for cancer chemotherapy, many Aurora kinase inhibitors have been discovered, and are currently under development. The binding modes of Aurora kinase inhibitors to Aurora kinases share specific hydrogen bonds between the inhibitor core and the back bone of the kinase hinge region, while others parts of the molecules may point to different parts of the active site via noncovalent interactions. Currently there are about 30 Aurora kinase inhibitors in different stages of pre-clinical and clinical development. This review summarizes the characteristics and status of Aurora kinase inhibitors in preclinical, Phase I, and Phase II clinical studies, with particular emphasis on the mechanisms of action and resistance to these promising anticancer agents. We also discuss the validity of Aurora kinases as oncology targets, on/off-target toxicities, and other important aspects of overall clinical performance and future of Aurora kinase inhibitors.

## Introduction

In 1995, David Glover discovered a new family of mitotic kinases while studying mutant alleles associated with defective spindle pole organization in *Drosophila melanogaster.* This family of kinases, which has been highly conserved during evolution, became known as the Aurora kinases (AKs) [1]. Humans have three homologous AKs, designated A, B and C. AKs are nuclear proteins, but they each have different sub-cellular locations. Aurora A is localized at the centrosome from the time of centrosome duplication through to mitotic exit [2, 3]. Aurora B, which is also known as the chromosomal passenger protein, is localized to the centromeres from the prophase to the metaphase-anaphase transition. Thereafter, it is localized to midzone spindle microtubules during telophase and subsequently to midbody during cytokinesis [2, 3]. Aurora C is also a chromosomal passenger protein considered to have a similar sub-cellular location to Aurora B. Aurora C is localized to centromeres during the prophase to metaphase and is redistributed to midzone microtubules during anaphase [4].

AKs are known to play multiple roles in mitosis, and their distribution correlates strongly with their functions. Aurora A is involved in mitotic entry, separation of centriole pairs, accurate bipolar spindle assembly, alignment of metaphase chromosomes, and completion of cytokinesis [5]. Recently, the role of Aurora A in the promotion of nuclear envelope breakdown has been described [6]. Aurora B is involved in chromosomal bi-orientation, regulating the association between kinetochores and microtubules, and cytokinesis [7]. Aurora B is also involved in the release of abnormal kinetochore microtubule attachments during chromosomal bi-orientation [8]. Aurora B is known to phosphorylate Histone H3 (Ser10), which then aids in chromatin condensation and separation [9]. It has been shown that Aurora C exhibits similar functions to those assigned to Aurora B and share the same substrates [10, 11].

Direct association with inner centromere protein (INCENP) activates Aurora C in vivo, which results in further complexation with Aurora B, suggesting the cooperation of Aurora B and C in the regulation of mitosis [10]. Like Aurora B, Aurora C associates with survivin and may be essential for cytokinesis. Wild-type Aurora C has also been reported to rescue multinucleation induced by enzymatically inactive Aurora B, indicating that Aurora C may complement the functions of Aurora B [11]. In summary, AKs play prominent roles in maintaining the genetic stability of cells. Aberrant expression of AKs leads to genomic instability or aneuploidy, hallmark of cancer cells [12].

## Aurora kinases as targets for cancer therapy

The Aurora A gene was originally named BTAK (breast tumor activated kinase) because its mRNA is overexpressed in breast tumors and it plays a critical role in the transformation of breast tumor cells [13]. Similarly, the Aurora A gene has been found to be amplified in human gliomas [14]. Using Northern and Southern blotting, Zhou et al. observed 2.5 to 8-fold amplification of Aurora A in many tumor cell lines [15]. Furthermore, Aurora A has been characterized as a potential low-penetrance tumor susceptibility gene, since the Phe31Ile functional polymorphism is strongly associated with familial breast cancer [16]. Similarly, Katayama et al. reported a correlation between overexpression of Aurora B and tumor progression in surgically resected colon tumor specimens [17]. The malignant progression of thyroid anaplastic carcinoma has also been shown to correlate with the overexpression of Aurora B [18]. The silent functional polymorphism, Ser295Ser (885 A > G) in the C-terminal end of Aurora B has been associated with an elevated risk of familial breast cancer [16], and overexpression of Aurora B has been correlated with decreased survival in glioblastoma patients [19].

In addition, aberrant expression of AKs has been shown to impair the functions of tumor suppressor genes, thereby generating aggressive tumors. Liu et al. reported that when overexpressed, Aurora A specifically phosphorylates p53 at Ser215 and inhibits its DNA binding and transcriptional activities [20]. Thus, inhibition of Aurora A may rescue the function of tumor suppressor genes.

Since AKs are aberrantly expressed in many cancer tissue types, and thereby generate aggressive tumors, they are regarded as important new-generation targets for cancer therapy.

## Small molecule Aurora kinase inhibitors (AKIs)

The discoveries of small molecule AKIs have been fuelled by the use of a variety of experimental and theoretical approaches. Examples include also structure-based drug design, especially in a fragment-based setup [21–24], structure-based virtual screening [25], FRET-based biochemical cell proliferation assay [26], and rational design followed by combinatorial expansion [27, 28].

**Fig. 1:**
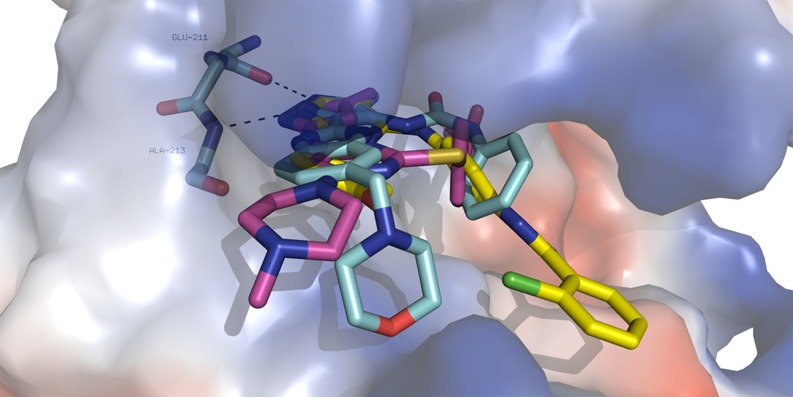
The crystallographic binding modes of three AKIs (in sticks, cyan—AT-9283, PDB (protein data bank) code 2W1E; yellow—bisanilinopyrimidine-based AKI, PDB code 3H0Y, violet—VX-680, PDB code 3E5A in the Aurora kinase A binding cleft (shown as surface). Specific hydrogen bonds to the backbone of residues Glu-211 and Ala-213 in the hinge region are shown by dotted lines. Color coding: oxygen—red, nitrogen—blue, chlorine—green, carbon—different colors. The figure was prepared using PyMol, ver. 0.99 [31]

Currently, more than 30 AKIs are in various stages of preclinical and clinical studies. Their core binds via specific hydrogen bonds to the hinge region of Aurora A [21, 29, 30]. The other parts of AKIs may span different regions of the active site and interact via various types of noncovalent interactions or stick to the solvent (Fig. 1). The interaction modes of two clinical compounds (AT-9283 and VX-680) and one bisanilinopyrimidine based preclinical AKI (Genentech, Aurora A: 3 nM IC50) have determined using PyMol, ver. 0.99 (Fig. 1).

In the following sections we discuss pan-Aurora kinase inhibitors (Table 1), the characteristics of specific inhibitors of Aurora A and Aurora B which are in clinical studies (Table 2), AKIs in advanced preclinical studies (Table 3), and finally AKIs in early preclinical studies and first generation AKIs (Table 4).

**Table 1:**
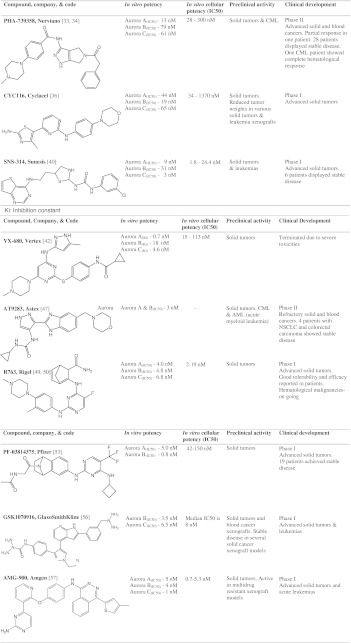
Pan-Aurora kinase inhibitors in clinical trials

**Table 2:**
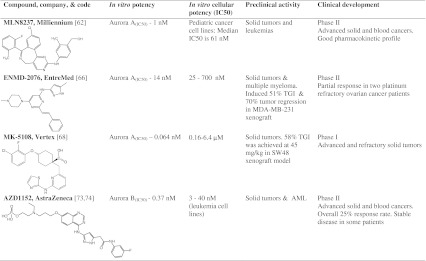
Aurora A or Aurora B inhibitors in the clinical trials

**Table 3:**
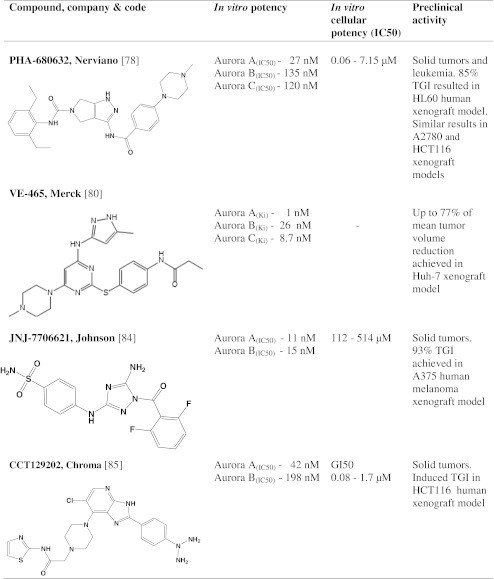
AKIs in advanced preclinical development

**Table 4:**
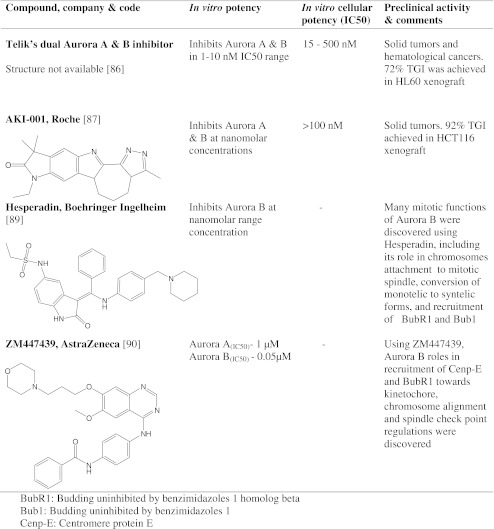
AKIs in early preclinical development and first generation AKIs

## Pan-Aurora kinase inhibitors in clinical trials

### PHA-739358

PHA-739358 (Danusertib), which was discovered and developed by Nerviano Medical Sciences, is currently in Phase II clinical studies. This inhibitor features a pyrrolopyrazole scaffold which had previously been identified as an ATP-mimetic pharmacophore suited for kinase binding [27]. The SAR (structure activity relationship) analysis of several pyrrolopyrazole subclasses resulted in the synthesis of PHA-680632 which showed high anti-cancer activity in vitro and in vivo and have thus become a preclinical candidate [27]. The X-ray crystallographic structure of PHA-680632 in complex with Aurora A guided further design. Through combinatorial expansion of a related 1,4,5,6-tetrahydropyrrolo[3,4-*c*]pyrazole core and SAR refinements of the 5-amido-pyrrolopyrazole series a potent Aurora kinase inhibitor PHA-739358 was identified [28]. It has been shown to inhibit Aurora A, B, and C in biochemical competitive assays with IC50 values of 13, 79, and 61 nM, respectively [28]. However, this study also demonstrated that PHA-739358 predominantly has an Aurora B inhibition phenotype in cell cultures. At high concentrations, it has been reported to cross-react with Abl (Abelson), Ret (rearranged during transfection), Trk-A, and FGFR1 (fibroblast growth factor receptor 1) kinases [32]. In this latter study, cell lines exposed to PHA-739358 were found to be sensitive to concentrations in the range 28 to 300 nM and the mode of action of PHA-739358 corresponded to Aurora B inhibition as assessed by phospho-histone H3(Ser10) inhibition. In addition, cells with tetraploid (4n) and polyploid (>4n) DNA content were observed to accumulate upon treatment with PHA-739358 [32]. Preclinical efficacy and toxicity studies were also performed in nude mice transplanted with several human xenografts, employing maximum tolerated doses (MTD) of 60 mg/kg/day for 5 days, 30 mg/kg/day for 10 days, or 45 mg/kg/day for 10 days. Tumor growth inhibition (TGI) was observed to be 66% to 98%; the compound was fairly well tolerated with only mild weight loss and myelosuppression. PHA-739358 has also been tested in a rat model having DMBA (9,10-Dimethylbenz-A-Anthracene) induced mammary carcinomas. At 25 mg/kg, TGI was measured as 75% and a complete cure was achieved in one rat [32]. Recently a Phase I study results were reported. Pharmacokinetic profiles were linear, and dose and time dependent. Of 80 patients assessed, stable disease was observed in 28, and in seven cases, this lasted for six months [33]. In another Phase I study, 56 patients divided into two parts (part 1 has 40 patients and part 2 has 16 patients) received escalating doses (45, 90, 180, 360, 500, 580, 650 mg/m^2^: 24 h infusion every 14 days) of PHA-739358 [34]. In part 1, patients received escalating doses of PHA-739358 without the co-administration of G-CSF (granulocyte stimulating factor). Doses were further escalated in part 2 in the presence of G-CSF. The MTD established in part 1 was 500 mg/m^2^. DLTs (dose limiting toxicity) were reported in 6 patients, which include neutropenia, grade 4 mucositis, and neutropenic infection. In part 2, 16 patients received the escalating doses of 500, 750, and 1000 mg/m^2^ along with G-CSF. No severe DLTs in the presence of G-CSF were reported even at maximum dose administered (1000 mg/m^2^). The dose 1000 mg/m^2^ of PHA-739358 along with G-CSF induced objective response in one refractory small cell lung cancer patient. This is the first time that the partial responses have been reported for an AKI with minimum toxicities. Several prolonged disease stabilizations were also reported in part 1 schedule. Phase II and Phase III single agent studies without G-CSF are underway in 7 types of solid tumors [34]. However, G-CSF is also being considered in further clinical studies. In addition to AKs, PHA-739358 has been also shown to inhibit BCR-ABL kinase (breakpoint cluster region-abelson) [35]. Many chronic myeloid leukemia (CML) patients acquire resistance to the BCR-ABL inhibitor imatinib by specific BCR-ABL mutations, particularly the T315I gate-keeper mutation. Interestingly, PHA-739358 inhibited both wild type BCR-ABL (25 nM) and T315I mutated protein in kinase assays. Moreover, PHA-739358 reportedly has a higher affinity for the T315I form (5 nM) than the Abl wild type (21 nM) [35], which may prove advantageous for clinical treatment. This compound is currently in Phase II studies, being investigated in imatinib-resistant CML patients [33]. Twelve CML patients were enrolled and received doses from 250 to 400 mg/m^2^/day (3 consecutive weeks every 4 weeks). Two patients with T315I BCR-ABL achieved complete hematological response. One patient displayed complete cytogenetic and molecular response after 3 months. PHA-739358 was well tolerated and only grade 3/4 neutropenia has been reported. As part of the pharmacodynamic study, CRKL phosphorylation was decreased in majority of treated patients. Additional studies in CML resistant patients are underway [33].

### CYC116

**Fig. 2:**
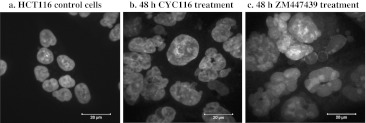
Confocal microscopic images of HCT116 colorectal cancer cells treated with CYC116 and ZM447439. a) DAPI (4′,6-diamidino-2-phenylindole) staining of diploid HCT116 parent cell line. b) & c) CYC116 and ZM447439 treatments resulted in the formation of polyploid cells

CYC116, discovered by Cyclacel Ltd., is an orally available AKI that has been tested in a Phase I trial. CYC116 was designed from the subset of lead N-phenyl-4-(thiazol-5-yl)pyrimidin-2-amines through cell-based screening of kinase-directed compound library [36]. The potency and specificity of CYC116 to Aurora kinases A and B was rationalized using X-ray crystal structure of the CDK2/CYC116 complex and docking to Aurora A structural model; indeed, specific residues responsible for the (differential) activity were identified [36]. It has been found to inhibit Aurora A, B, and C with IC50 values of 44, 19, and 65 nM, respectively, and also VEGFR2 (vascular endothelial growth factor receptor 2) with an IC50 of 69 nM [37]. In this study, the proliferations of various cancer cell lines with different genetic backgrounds were inhibited with IC50 values of 34 to 1370 nM. CYC116 is a targeted drug that has antimitotic and anti-angiogenesis properties [36]. It was shown to inhibit autophosphorylation of Aurora A and B in A549 lung cancer cell lines, demonstrating its specificity against AKs, and it also induced failed mitosis, resulting in polyploidy (Fig. 2), which eventually killed the cells by apoptosis [36]. Further, CYC116 exhibited antitumor activity in various leukemia, solid tumor xenograft and leukemic syngenic models [36]. In mice with P388/D1 leukemia, it suppressed angiogenesis, decreased phosphorylation of histone H3, and induced accumulation of 4n and >4n DNA in cells [37]. It was reported to significantly reduce tumor neovascularization in a dose-dependent manner, possibly due to inhibition of VEGFR2 [38]. In P388D1 mouse leukemia model, at 45 and 67 mg/kg twice daily, the drug increased the life span of 172% and 183%, respectively. Oral administration of CYC116 in NCI-H460 xenograft, at 75 and 100 mg/kg for 5 days caused significant tumor growth delays. Adverse side effects have not been reported. A Phase I trial in advanced solid tumors has been conducted to determine its MTD and evaluate its pharmacokinetic properties [36].

### SNS-314

SNS-314 is a pan-Aurora kinase inhibitor discovered and developed by Sunesis pharmaceuticals which has been tested in a Phase I clinical trial. It was designed from the lead molecule, 2-aminoethyl phenyl benzamide through structure-activity optimizations. It has been reported to inhibit Aurora A, B, and C with IC50s of 9, 31, and 3 nM, respectively [39]. Additionally, it was shown to inhibit 24 other kinases with higher IC50 values. It inhibited cell proliferation in different human cell lines with IC50 values of 1.8 to 24.4 nM, and induced polyploidy. Histone H3 phosphorylation was significantly inhibited in all 6 cell lines tested with IC50 values 9–60 nM. The anti-tumor activity of SNS-314 was tested in several solid tumor xenograft models [39]. Preliminary in vivo study to determine dosing and schedules was performed in HCT116 xenograft. This study involved administration on a biweekly schedule for three weeks and reported 54–91% TGI at 170 mg/kg in breast, prostate, lung, ovarian, and melanoma xenografts. Single dose of SNS-314, 50 or 100 mg/kg, led to the complete inhibition of histone H3 phosphorylation as early as 3 h after administration in HCT116 xenograft model. This corresponded to the appearance of polyploid cells and caspase-3 activation [39]. The drug has been subjected to a Phase I clinical trial involving 32 advanced solid tumor patients, divided into 8 cohorts with doses ranging from 30 to 1800 mg/m^2^ [40]. Only Grade 1 and 2 toxicities were observed, suggesting it was well tolerated. A dose limiting toxicity, namely neutropenia (Grade 3), was observed at a dose of 1440 mg/m^2^. Plasma levels of SNS-314 were dose-proportional and no drug accumulation was reported. Stable disease has been reported in 6 patients with advanced solid tumors [40].

### VX-680

VX-680 was discovered by Vertex Pharmaceuticals, Oxford, UK. It was designed during the SAR exploitation of a lead molecule amino pyrazole linked to 2-substituted quinazoline [41]. It has been shown to inhibit Aurora A, B, and C with Ki values of 0.7, 18, and 4.6 nM, respectively [42]. In cytotoxicity assays with several tumor cell lines, VX-680 was reported to inhibit proliferation with IC50 values ranging from 15 to 130 nM. It was also observed to disrupt mitosis in a wide variety of tumor cell lines by affecting chromosome segregation and cytokinesis, eventually inducing the accumulation of cells with 4n DNA content, activating checkpoints, and subsequently inducing apoptosis [42]. Despite promising results, clinical trials involving this compound were suspended due to its toxicity profile (one case of heart failure). However, the compound has been tested in patients with advanced CML, acute lymphoblastic leukemia (ALL), and myelodysplastic syndromes because it has been found to successfully inhibit the T315I mutated form of BCR-ABL, which is resistant to imatinib. This surprising result prompted a re-examination of other clinical compounds against drug-resistant kinases. VX-680 has been observed to bind to wild type BCR-ABL with a dissociation constant *K*_d_ of ~20 nM and to T315I (as well as other Abl mutants) with a *K*_d_ of 5 nM. In vitro assays have shown that VX-680 inhibits the activity of wild type BCR-ABL and BCR-ABL (T315I) with IC50 values of 10 and 30 nM, respectively [43].

### AT9283

AT9283 developed by Astex Therapeutics is the first AKI discovered through the company’s proprietary fragment-based screening approach. The pyrazole-benzimidazole hit compound was improved by SAR to a lead. The lead optimization was guided by X-ray crystallography and finally resulted in AT9283 as a clinical candidate [21]. It is currently in several Phase II studies under the Cancer Research UK. It has been shown to be an inhibitor of several kinases, including Aurora A (3 nM), Aurora B (3 nM), JAK2 (janus kinase) (1.2 nM), JAK3 (1.1 nM), and Abl (4.0 nM, T315I) [21]. HCT116 cells exposed to AT9283 exhibited polyploid phenotypes, which are typically associated with predominant Aurora B inhibition [21]. Like PH3-739358, AT9283 was found to inhibit wild-type BCR-ABL and T315I BCR-ABL (IC50 values of 110 and 4 nM, respectively) [44]. AT9283 has also been observed to inhibit proliferation of BaF3 cells with both wild type BCR-ABL and T315I BCR-ABL (IC50 values of 13 and 11 nM, respectively). In cellular assays, it inhibited the proliferation of BCR-ABL driven chronic myelogenous leukemia (BaF3) cells as judged by the inhibition of CRKL (v-crk sarcoma virus CT10 oncogene homolog (avian)-like) phosphorylation. In vivo efficacy of AT9283 was tested in BaF3 xenograft with either BCR-ABL wild type or T315I mutation. At 12.5 mg/kg for 5 days, followed by 2-day drug holiday AT2983 significantly inhibited tumor growth without severe toxicities. It has also been found to induce significant reductions of tumor volume in K562 (CML, BCR-ABL positive) xenograft mouse model at 12.5 mg/kg [44].

In one trial, skin punch biopsies were taken for subsequent immunohistochemical studies from patients treated with AT9283, as well as serum samples collected at regular intervals. Inhibition of histone H3 phosphorylation, p53 stabilization, reduction of PCNA and Ki67 were detected. Analysis of the serum samples indicated elevation of M30 and M65 apoptotic markers and caspase activation [45]. The safety, tolerability, and preliminary efficacy of this compound are currently being evaluated in Phase I/II clinical studies. In one study, 30 patients with different refractory leukemias were enrolled for part of a Phase I trial. Patients were treated with escalating doses of AT9283, rising from 3 to 162 mg/m^2^/day [46]. No DLT was observed at doses below 72 mg/m^2^/day, except in one patient who developed tumor lysis syndrome at 12 mg/m^2^/day. At the maximum administered dosage, two DLTs and three deaths were reported, so further attention was focused on sub-maximum doses. Commonly observed DLTs included septicemia, pneumonia, and mucositis [46]. In a second study, 40 patients (five cohorts) with refractory solid tumors were enrolled as part of a second Phase I clinical study. Patients were treated with escalating doses, rising from 1.5 to 12 mg/m^2^/day. No DLTs were observed at doses below 6 mg/m^2^/day. At 12 mg/m^2^/day, two patients developed febrile neutropenia; 9 mg/m^2^/day was identified as the MTD. At this dosage, 3% of patients showed a partial response and 30% displayed stable disease [47].

### R763

R763 (AS703569), which was discovered and developed by Rigel, is an orally available Aurora inhibitor, currently in Phase I study. It was designed and developed based on a image-based phenotypic screen. It has been reported to inhibit Aurora A, B, and C with IC50 values of 4, 4.8, and 6.8 nM, respectively and to inhibit Abl, FLT1 (fms-related tyrosine kinase), and FLT3 oncogenic kinases [48]. In this study, Colo205, MiaPaCa-2, HeLa, and MV4-11 cells were observed to be most sensitive to R763 (IC50 = 2 to 8 nM), but primary proliferating cells were also sensitive despite having higher IC50 values (IC50 = 31 to 160 nM). This could be due to slower cell proliferation and intact cell cycle checkpoints. No effect was observed on non-dividing cells at the highest concentration tested. R763 appeared to induce endoreduplication within 48 h as evidenced by the accumulation of 4n and 8n cells. Colo205, HeLa, and MiaPaCa-2 underwent apoptosis after 48 h. In a preclinical phase, the in vivo efficacy of R763 was tested in MiaPaCa-2, adriamycin-resistant tumor, MOLT-4, and MV4-11 xenograft models. Significant reduction in tumor volumes did not occur in the MiaPaCa-2 xenograft model, but histological regression and reduction in histone H3 phosphorylation (Ser10) was observed. In contrast, tumor volumes were significantly reduced in adriamycin-resistant tumors. Treatment of the MOLT-4 xenograft model resulted in a 5–10% reduction in the total number of bone marrow cells. The percentages of leukemia cells were significantly reduced, whereas control groups were not affected. In the MV4-11 xenograft, R763 induced pronounced anticancer activity in a dose-dependent manner. For a dose of 20 mg/kg/day, undetectable levels of tumors were seen in 17% of animals. Increased life span was observed in all treated groups, whereas all control mice died early [48].

Two Phase I studies were completed and one study is underway. Data from two studies were reported at international meetings. Initial clinical studies have been performed with two different dosing regimens to determine the compound’s MTD, toxicity, and pharmacodynamic profile [49]. Cohorts of three patients were assigned to one of the two regimens. The starting dose was 6 mg/m^2^ *p.o.* (Per Os) per 21-day cycle divided into two or three doses. Regimen 1 involved dosing on days 1 and 8, while regimen 2 involved dosing on days 1, 2, and 3. 15 patients were enrolled, including three with uterine, three with cervical, and two with breast cancer. Initially, two cohorts of three patients were treated at dose level 1, and no significant toxicity or adverse side effects were observed. Two patients did not receive effective treatment and one patient withdrew consent. During this study, two patients received 4+ dosing cycles and one received 3+. Overall, both dose levels (6 and 12 mg/mg^2^) were well tolerated [49]. Further dose escalations were carried out in patients with hematological malignancies in a second Phase I study [50]. Two dosing regiments were tested: days 1–3 and 8–10 of a 21-day cycle (regimen 1) and days 1–6 of a 21-day cycle (regimen 2). In regimen 1, 24 patients were treated up to dose levels of 47 mg/m^2^. At the maximum dose of 47 mg/m^2^, two grade 3 diarrheas have been reported. In regiment 2, 21 patients were also treated up to dose levels of 47 mg/m^2^. At this dose two DLTs namely, one neutropenic infection and two grade 4 mucositis have been reported. In this Phase I study, the established MTD was 37 mg/m^2^. Other reported toxicities include neutropenia, anemia, thrombocytopenia, and gastrointestinal disorders. One patient with CML (T315I) displayed hematological and cytogenetic response, one CML patient had a partial response, three AML patients achieved reduction in peripheral blasts, and several disease stabilizations were also reported [50]. Further enrollment of patients was ongoing at the time of report. Another Phase I study of R763 in combination with gemcitabine in advanced malignancies was recently completed.

### PF-03814735

Pfizer’s PF-03814735 is another orally available dual Aurora-A and Aurora-B inhibitor, which is currently in a Phase I study. It was discovered by SAR exploitation of lead pyrimidine scaffold. PF-03814735 was eventually designed by SAR optimizations at C2 and C4 positions of pyrimidine scaffold [51]. It has been found to inhibit recombinant Aurora A and Aurora B with IC50 values of 5 and 0.8 nM, respectively, as well as FLT1, FAK (focal adhesion kinase), TrkA, MET, and FGFR1 kinases at higher IC50 values [52]. It has also been shown to inhibit the proliferation of various human tumor cell lines (IC50 = 42 to 150 nM). In this study, phosphorylation of Aurora B (Thr232) was reduced significantly in MDA-MB-231, using a concentration of PF-03814735 close to the IC50 (about 20 nM). It was also found to inhibit phosphorylation of histone H3 (Ser10) with an IC50 value of ~50 nM. Aurora A autophosphorylation (Thr288) was also inhibited at an IC50 value of ~150 nM, which is 3-folds higher than histone H3 (Ser10) phosphorylation inhibiton. PF-03814735 was shown to inhibit Aurora A and Aurora B rapidly and reversibly in cell cultures. When HCT116 cells were treated with PF-03814735, initially 4n DNA content cells accumulated followed by ≥8n DNA content, consistent with failed mitosis. At similar concentrations, inhibition of phospho-histone H3 was observed in athymic mice bearing HCT116 xenograft. Mice bearing HCT116 tumors were treated once daily with 10, 20, or 30 mg/kg for 10 days. Significant tumor growth inhibition (≥50%) occurred at ≥20 mg/kg. Moreover, significant antitumor efficacy was observed when PF-03814735 was tested in A2780, MDA-MB-231, Colo-205, and HL-60 xenograft models. Mice xenograft models tolerated various dosing schedules with very few toxic effects [52]. In Phase I initial clinical study, 57 patients with solid tumors were treated [53]. In schedule A, 32 patients received 5–100 mg/day for 5 days; or in schedule B patients (25) received 40–60 mg/day for 10 days of 21-day cycles. The MTD for schedule A was 80 mg/day. One patient in schedule A experienced grade 3 proctalgia and two patients experienced grade 3 and grade 4 febrile neutropenia. The MTD for schedule B is 50 mg, where two patients experienced grade 3 increase of aspartate aminotransferase and grade 2 ventricular dysfunction. PF-03814735 was rapidly absorbed, appeared in circulation within 6 h of dosing, and it exhibited favorable linear pharmacokinetics. Pharmacodynamics of PH-03814735 was evaluated using phospho-histone H3 (Ser10) staining of mitotic cells as a surrogate biomarker. In comparison to the baseline, phospho-histone H3 levels decreased in 10 patients and paradoxically increased in two treated patients. In terms of efficacy, 19 patients achieved stable disease. Moreover in schedule A, five patients with stable disease displayed low tumor shrinkage [53].

### GSK1070916

GlaxoSmithKline’s GSK1070916 is a reversible Aurora B and C inhibitor that is currently studied by Cancer Research UK in a Phase I clinical study. It was designed from the various SAR refinements of a lead 7-azaindole series [54]. It has been shown to inhibit Aurora B-INCENP and Aurora C-INCENP with IC50 values of 3.5 and 6.5 nM, respectively, and to cross-react with FLT1, TIE2 (tyrosine kinase with immunoglobulin-like and EGF-like domains 1), SIK (salt inducible kinase), FLT4, and FGFR1 at higher concentrations [55]. The in vitro activity of GSK1070916 has been tested on 161 tumor cell lines and found to inhibit the proliferation of cancer cell lines with a median IC50 of 8 nM [56]. It did not show any potent anticancer effects on non-proliferating HUVEC cells (IC50 = 3900 nM). In A549 cell line, it induced polyploidy and apoptosis in a dose dependent manner, which is consistent with Aurora B inhibition. Apoptotic cell death was evidenced by induction of caspase-3 and PARP cleavage in Colo205 cells. In vivo efficacy was tested in several xenograft models at 25, 50, or 100 mg/kg once daily for five consecutive days, followed by two days off, for two or three cycles. Complete or partial regressions were achieved in A549, HCT116, HL60, and K562 xenograft models and stable disease was observed in Colo205, H460, and MCF-7 xenografts. Adverse toxicities were not reported for this compound. Its efficacy was also tested in two human leukemia models: MV-4-11 and HL60. Significant dose-dependent increase in median survival times were reported [56]. GSK1070916 Phase I clinical study is currently recruiting patients with advanced solid tumors.

### AMG-900

Amgens’s AMG-900 is an orally available pan-Aurora kinase inhibitor that is currently in Phase I clinical studies. It has been shown to inhibit Aurora A, B, and C with IC50 values of 5, 4, and 1 nM, respectively [57]. It has also been shown to cross-react with other kinases including p38α, TYK2 (tyrosine kinase 2), JNK2, MET, and TIE2 with IC50 values in the range of 53–650 nM. It has been found to inhibit the proliferation of 26 diverse cancer cell lines with IC50 values between 0.7–5.3 nM. Interestingly it was able to overcome the resistance in PgP (P-glycoprotein) expressing multidrug resistant cancer cell lines, as it inhibited colony formation of resistant and parent cell lines uniformly. Strikingly other AKIs (AZD1152, VX-680, PHA-739358) were less potent than AMG-900, when tested on these multidrug resistant cell lines. Moreover, the compound was also shown to inhibit AZD1152 resistant HCT116 cell line harboring Aurora B mutation (W221L). AMG-900 inhibited both parent and AZD1152 HCT116 resistant cell lines with equal potencies in colony formation assay [57], while human foreskin fibroblasts were relatively insensitive to the drug. However, it induced cell death in proliferating human bone marrow mononuclear cells at nanomolar concentrations, suggesting its high activity in cycling cells. AMG-900 inhibited autophosphorylation of Aurora A (Thr288) and histone H3 phosphorylation (Ser10) in a dose-dependent manner, with IC50 values of 6.5 and 8.2 nM, respectively. AMG-900 predominantly showed Aurora B inhibition phenotype, as evidenced by the appearance of polyploid HeLa cells due to failed cytokinesis. Appearance of polyploid cells corresponded to the induction of p53 and p21 levels, which are widely accepted biomarkers related to Aurora inhibition. The compound induced time-dependent induction of apoptosis, as evidenced by the increase in the caspase-7 levels over the period of time. In HCT116 xenograft model it inhibited histone H3 phosphorylation in a dose-dependent manner. As expected it also suppressed histone H3 phosphorylation in mouse bone marrow cells. Treatment of mice with AMG-900 at 3.75, 7.5, and 15 mg/kg/twice daily for 2 consecutive days/week/3 weeks resulted in dose-dependent TGI’s in the range 40 to 75%. Toxicities reported include moderate weight lose and myelosuppression. Dose-dependent TGI’s were also reported in an alternate daily dosing schedule. It has also been tested in other xenograft models and 3 multidrug resistant (MDR) xenograft models. Two schedules were employed for this study. The xenografts were treated at 15 mg/kg b.i.d/2 consecutive days/week or 3 mg/kg b.i.d/day. AMG-900 exhibited significant antitumor activity (50–97% TGI) in all the xenografts models including MDR xenografts. It was able to overcome the drug resistance of MDR xenografts, otherwise insensitive to docetaxel or paclitaxel at their respective MTDs. Importantly, inhibition of histone H3 phosphorylation correlated with plasma drug concentrations [57]. Overall AMG-900 displayed favorable pharmacokinetic and pharmacodynamic profiles with anticipated minimum toxicities. Importantly, AMG-900 has great potential to overcome both the tumor multidrug resistance and to show activity in cancers resistant to other AKI due to mutation of the Aurora kinase B binding site. Currently two Phase I studies are underway in patients with advanced solid tumors and acute leukemias.

## Aurora A inhibitors in clinical trials

### MLN8237

MLN8237 (Alisertib) discovered by Millennium pharmaceuticals has been reported to be a highly specific and potent inhibitor of Aurora A (IC50 = 1 nM) [58]. This is a second generation Aurora A inhibitor from this company, as the predecessor to MLN8054. MLN8054 was terminated in Phase I studies due to off-target toxicities observed. This led to the development of new Aurora A specific inhibitor by the company, with a code name, MLN8237. MLN8237 was designed through SAR optimization of lead 5-H-pyrimido[5,4-d][2]benzazepine. It is currently in numerous Phase II clinical studies. It does not appear to have any significant off-target effects towards other kinases included in the panel, but it has been shown to inhibit wild-type BCR-ABL and T315I BCR-ABL effectively in both kinase assays and in vitro cell cultures [59]. It has also been found to inhibit growth in HCT116, PC3, SK-OV-3, and LY-3 cancer cells lines in cell proliferation assays, with GI50 values between 16 and 469 nM [58]. The specificity of MLN8237 has been tested in multiple myeloma (MM) cell lines [60]. In this study, autophosphorylation of Aurora A (Thr 288) was markedly inhibited at 0.5 μM in MM cell lines. MLN8247 induced 2 to 6-fold accumulation of G2/M followed by apoptosis, as evidenced by cleavage of PARP, caspase-9, and caspase-3. In addition, cell death by senescence was predominant after long exposure of MM cell lines. The efficacy of MLN8237 was tested in vivo in a MM xenograft model implanted in SCID (severe combined immune deficiency) mice. Tumor volumes were found to be significantly reduced at 30 mg/kg, and TGI was determined to be 42% and 80% at 15 and 30 mg/kg, respectively. Further, the overall survival rates of animals were significantly prolonged [60]. MLN8237 has also been tested on many pediatric cancer cell lines including rhabdomyosarcoma, Ewing sarcoma, glioblastoma, neuroblastoma, ALL, and AML [61]. In this study, the median IC50 was reported as 61 nM, ALL cell lines displaying the highest sensitivity and rhabdomyosarcoma cell lines were the least sensitive. Disease-free survival was improved in 80% of solid tumor models and 100% in ALL models, even more promising, a sustained complete response was achieved in 3 of 7 neuroblastoma models and the activity was much higher than standard anticancer agents [61]. Phase I dose escalation and dose-limiting toxicity studies involving cohorts of three patients with advanced solid tumors have been completed [62]. Each patient was given an oral dose once per day for seven days in a 21-day cycles, with the dosage increasing from 5 to 150 mg/day until DLTs were observed in more than two patients. DLTs were not reported for doses of 5–80 mg/day. However, in some patients mixed DLTs were reported at 150 mg/day, including G3 and 4 neutropenia, G3 somnolence, G3 mucositis or oral candidiasis, confusion, agitation, and alopecia. Aurora A kinase inhibition was inferred from the accumulation of mitotic cells in skin and tumor biopsies [62]. Plasma levels of MLN8237 were found to be dose-proportional, suggesting MLN8237 has a good pharmacokinetic profile. Currently multiple Phase II MLN8237 studies are recruiting patients with a wide range of solid cancers and blood cancers for optimal dosing regimen, efficacy, and MTD determination.

### ENMD-2076

EntreMed’s ENMD-2076, currently in Phase II clinical trials, has been shown to selectively inhibit Aurora A with an IC50 of 14 nM measured in biochemical assays [63]. The molecule was designed by SAR optimization of a lead imidazole-vinyl-pyrimidine scaffold. It was also found to inhibit multiple oncogenic kinases, namely FLT3 (3 nM), Src (sarcoma) (20 nM), VEGFR2 (36 nM), and FGFR1 (93 nM), as well as the growth of various cancer cell lines (IC50 = 25 to 700 nM) [63]. It was observed that 5 μM ENMD-2076 induced G2/M arrest in HCT116 cells consistent with Aurora A inhibition, rapidly inducing apoptosis. Recently, ENMD-2076 has also been shown to be highly effective against MM cell lines and primary MM cells derived from patients [64]. In this study, ENMD-2076 was found to cause 50% cell death in MM cell lines at a concentration of 3 μM for 72 h of exposure. It also induced apoptotic cell death after only 6 h of exposure as evidenced by annexin-V staining, PARP cleavage, and activation of caspase-9, 8, and 3. In MM cells it significantly reduced autophosphorylation of Aurora A (Thr288) after 24 h of exposure. However, it also inhibited Aurora B at concentrations that resulted in cell death, as shown by down-regulation of histone H3 phosphorylation (Ser10). The in vivo efficacy was tested in a H929 human plasmocytoma xenograft model at doses of 50, 100, and 200 mg/kg. A dose-dependent efficacy was observed in all animals and maximum affect was achieved at 200 mg/kg with good tolerability. Immunohistochemistry on sacrificed animals revealed reduced Ki67 levels, increased caspase-3 levels, and decreased phospho-histone levels in treated animals compared to an untreated control [64]. When a HT-29 xenograft model was dosed at 100 or 200 mg/kg once per day, the tumor volumes remained static until around 17 days, and moderate regression was subsequently observed for 200 mg/kg. Immunohistochemistry revealed there was a corresponding reduction in Ki67 levels. ENMD-2076 has also been tested in a patient-derived colorectal cancer (CRC) xenograft where it was found to induce TGI in all cases (K-ras mutant) [65]. In the MDA-MB-231 mouse xenograft model, ENMD-2076 has been observed to reduce tumor growth by 51% at a dose of 50 mg/kg per day and to cause tumors to regress by 70% at a dose of 200 mg/kg [63]. Recently, Phase I study results were reported including pharmacokinetic, pharmacodynamic, and antitumor activity profiles. Patients with refractory advanced solid tumors were treated with continuous oral daily dosing. Doses in the range 60 to 200 mg/m^2^ were tested in a standard 3 + 3 design. Totally 67 patients were enrolled for this study [66]. At 200 mg/m^2^, two patients displayed grade 3 hypertension and 160 mg was reported as MTD. ENMD-2076 has linear pharmacokinetic profile and displayed significant antitumor activity including decreased VEGFR2 levels in plasma. The highest activity was reported in ovarian cancers, where two patients with platinum refractory disease showed partial responses [66]. Three Phase I studies are currently underway being tested in advanced solid tumors and multiple myeloma.

### MK-5108

MK-5108 (VX689), discovered and developed by Vertex Pharmaceuticals, has been studied in a Phase I clinical trial in patients with advanced solid tumors. It has been shown to inhibit Aurora A with an IC50 value of 0.064 nM [67]. It also inhibited Aurora B and Aurora C at higher IC50 values (220 and 190 folds higher than Aurora A). It has been shown to inhibit the proliferation of 17 diverse cancer cell lines with IC50 values ranging from 0.16 to 6.4 μM. MK-5108 significantly enhanced the efficacy of docetaxel in HeLa-S3 and ES-2 cell lines. MK-5018 and docetaxel combination also showed similar efficacy in HeLa-luc and ES-2 xenograft models. In cell lines it predominantly showed Aurora A inhibition phenotype (G2/M arrest), as histone H3 phosphorylation was not inhibited, which is a marker for Aurora B inhibition. MK-5108 inhibited Aurora A, as also evidenced by the inhibition of Aurora A autophosphorylation (Thr288). MK-5108 induced greater accumulation of phospho-histone H3 at much lower concentrations compared to MLN8054, a well known Aurora A specific inhibitor [67]. In vivo efficacy of MK-5108 was tested in HCT116 and SW48 xenograft models. Doses of 15 and 30 mg/kg were administered twice daily for 12 days. MK-5108 treatment resulted in TGI’s of 10% and 17% at doses 15 mg/kg and 30 mg/kg, respectively in HCT116 xenograft model. In SW48 xenograft model, intermittent doses (twice daily/2 days/week/3 weeks) of 15 mg/kg and 45 mg/kg resulted in 35% and 58% TGI’s, respectively. MK-5108 was well tolerated and adverse toxicities were not reported.

MK-5108 was tested in patients with advanced solid tumors either as a single agent or in combination with docetaxel. Febrile neutropenia and myelotoxicity were reported as DLTs in the combination treatment regimen. However, no significant toxicities were reported in the monotherapy arm. Disease stabilization was reported in 32% patients from both arms and partial responses were reported in 12% of patients only from the combination arm [68].

## Aurora B inhibitors in clinical trials

### AZD1152

AZD1152 (Barasertib) is an AstraZeneca compound which has been shown to be a highly specific inhibitor of Aurora B (0.37 nM) [69], and is currently in Phase II clinical studies. It was designed and developed from the lead pyrazole-acetanilide-substituted quinazoline by SAR exploitation [70]. It has been found to exhibit varying potency across different types of leukemia cells (ALL, PALL-2, MOLM13, MV4-11) inhibiting their proliferation with IC50s ranging from 3 to 40 nM, and also inhibits the proliferation of freshly isolated patient leukemia cells (IC50 = 3 nM). In this work, exposure of MOLM13 and PALL-2 to AZD1152 resulted in accumulation of 4n/8n DNA cells, which subsequently underwent apoptotic cell death as demonstrated by annexin-V staining [69]. In SW620 colon cancer cells, it has been observed to inhibit the phosphorylation of histone H3 in a dose-dependent manner, which is indicative of Aurora B inhibition [71]. Furthermore, it caused potent dose-dependent growth inhibition of human xenograft models in nude mice, including SW620, HCT116, Colo205, A549, Calu-6, and HL-60. In these experiments, the extent of growth inhibition ranged from 55 to 100%; the HL-60 model was the most responsive (complete regression was observed). Elevated caspase-3 levels were observed in all tumors isolated for histological assessment. The mechanism of AZD1152 action was found to be similar in both in vitro and in vivo conditions. AZD1152 was well-tolerated at doses required for efficacy; myelosuppression is the primary problem associated with high doses [71]. Treatment of the human MOLM13 xenograft immunodeficient murine model with AZD1152 at a dose of 25 mg/kg per day caused significant reductions in tumor weight and growth [69]. However, none of the mice showed any signs of side effects, which suggest that it was well tolerated.

Initial clinical study was conducted on 13 patients having colon cancer, melanoma or some other solid tumors. The compound was administered via intravenous (i.v.) infusion (2 hrs per week) in a dose-escalating manner (100–450 mg) on days 1, 8 and 15 of a 28-day cycle. Doses up to 300 mg were well tolerated, but neutropenia was observed in three patients at 450 mg. Significant disease stabilization was observed in progressive cancers [72]. AZD1152 recently entered Phase I/II clinical trials focusing on its safety, tolerability, pharmacokinetics, and efficacy profiles in AML patients [73]. Treatment of AML patients was performed in two parts; in part A, 32 individuals were treated (continuous 7-day infusion every 21 days) at doses ranging from 50 to 1600 mg. Grade 3/4 stomatitis or mucosal inflammation were reported as DLTs at doses ranging from 800 to 1600 mg. Most of the toxicities resolved following dose delay and no treatment related deaths have been reported. This part of the study established MTD as 1200 mg. Consequently, another 32 patients received 1200 mg in part B of the study. For combined part A and part B, the overall response rate was 25% in both newly diagnosed and relapsed AML patients. The pharmacokinetic profiles were favorable as assessed by the AZD1152 blood levels and distribution to tissues [73]. Recently, barasertib has been tested in patients with advanced solid malignancies using escalating doses from 100 mg to 650 mg per day [74]. In schedule A, 2 h i.v. infusion was given for every 7 days across four escalating doses (100, 200, 300, and 450 mg). In schedule B, the drug was administered every 14 days across five escalating doses (200, 300, 450, 550, and 650 mg). Schedule A included 19 patients and schedule B included 40 patients. Doses 250 mg and 400 mg per day were the MTDs in schedules A and B, respectively. Neutropenia and leukopenia were the most common side effects. Objective antitumor effects were not observed, however, stable disease achieved in 15 patients overall. In this study, the linear pharmacokinetics has also been reported, as the systemic exposure to AZD1152-HPQA (active drug) was observed by 1 h into the infusion [74]. Currently AZD1152 is being tested in a Phase II trial in large B-cell lymphoma patients.

### BI 811283

Boehringer-Ingelheim’s BI 811283 is a Aurora B inhibitor that is currently in a Phase II clinical study. It has been shown to inhibit Aurora B with IC50 value of 9 nM and also inhibited the proliferation of 24 diverse cancer cell lines with an IC50 value <14 nM [75]. Chemical structure of BI 811283 is not disclosed by the company. Treatment of cancer cell lines with BI 811283 resulted in polyploidy within 48 h due to failed mitosis. It dominantly induced senescence (based on SA-beta-GAL staining) within 96 h and only 7% cells showed apoptotic phenotype (PARP cleavage and nuclear fragmentation) after 96 h. In vivo efficacy of BI 811283 was tested in NSCLC and colorectal cancer cell line xenograft models. The compound was administered once weekly by 24 h s.c. infusion. It inhibited tumor growth of xenografts in dose-dependent manner and at the MTD (20 mg/kg), tumor regression was reported in some animals. Accumulation of lager and multinucleate cells were reported, which is consistent with the Aurora B inhibition phenotype [75].

In Phase I dose escalation study, BI 811283 has been tested in advanced and metastatic solid tumors [76]. Patients were randomized into two schedule treatment groups, q2w and q3w in a bicentric Phase I dose escalation. In 3-week treatment schedule, patients were treated with BI 811283 as 24 h i.v. infusion on day 1 of each 21-day treatment cycle. The MTD was reported to be 230 mg/kg. The main side effects include reversible hematotoxicity, neutropenia, and febrile neutropenia. However, accumulated toxicity was not reported in two patients that are treated for >16 courses. As part of the efficacy, stable disease was reported in 33.3% of patients [76]. In another 4-week treatment schedule, patients received BI 811283 (5–140 mg/kg) as 24 h i.v. infusion on days 1 and 15 of each treatment cycle [77]. The MTD was reported to be 140 mg/kg. The dose limiting toxicities were almost identical to previous schedule. Stable disease was reported in 29% of patients. Pharmacokinetic profiles were near-linear and the half-life was 11.9 to 26 h. Additonal dosing schedules in expanded patient cohorts were also completed. However, the results are not yet published. A Phase II clinical study in combination with cytarabine is currently underway.

## AKIs in advanced preclinical studies

### PHA-680632

PHA-680632 is another pan-Aurora kinase inhibitor from Nerviano. It emerged from SAR modifications of several pyrrolopyrazole core sub-classes of ATP-mimetic pharmacophores [27]. It has been reported to inhibit Aurora A, B, and C with IC50 values of 27, 135, and 120 nM, respectively, and to cross-react with FGFR1 (IC50 = 390 nM) [78]. It has also been shown to inhibit proliferation of various cancer cell lines with different genetic backgrounds (IC50 = 0.06 to 7.15 μM). Further, this study found that PHA-680632 selectively generated polyploidy in a cancer cell line—HCT116, but not in a normal cell line. Treatment of cells with anti-Aurora A siRNA, but not anti-Aurora B siRNA induced accumulation of active caspase 9 and 3. Similarly PHA-680632 induced accumualtion of active caspase 9 and 3, which is an indicative of predominant Aurora A inhibition related apoptosis. Treatment of HeLa cells with 2 μM PHA-680632 for 24 h resulted in dramatic down-regulation of phospho histone H3 (Ser10). Its efficacy and toxicity were tested in human tumor xenograft models, and in mouse and rat syngenic models. These tests involved administration of PHA-680632 at a dose of 45 mg/kg for five consecutive days to mice bearing the HL60 tumor and resulted in a TGI of 85% compared to tumor growth in control animals treated only with the vehicle. Similar effects were observed in A2780 and HCT116 models. In A2780 mouse xenograft model, inhibition of histone H3 phosphorylation was observed within 8 h of dosing at 60 mg/kg. No toxicities were reported at any of the doses employed [78]. Treatment of HCT116p53-/- cells with PHA-680632 after ionising radiation exposure (IR) has been shown to result in enhanced cell killing (with additive effect) as determined by annexin-V staining, micronuclei and BRCA-1 foci formation. Correspondingly, combined treatment of IR and PHA-680632 in HCT116p53-/- mice xenograft model showed enhanced tumor growth delay [79].

### VE-465

VE-465 is another pan-Aurora kinase inhibitor discovered by Vertex pharmaceuticals. The chemical structure is similar to that of VX-680. VE-465 was designed by SAR optimization of the lead amino pyrazole. Using ATP (adenosine triphosphate) competitive binding assays it has been shown to inhibit Aurora A, B, and C with Ki values of 1, 26, and 8.7 nM, respectively [80]. In preclinical studies it exhibited anticancer effects on two hepatocellular carcinomas, Huh-7 and HepG2. It also suppressed Aurora B activity in a dose-dependent manner within 1 h treatment of both cell lines. Immunocytochemistry studies in Huh-7 and HepG2 using anti α-tubulin and DAPI indicated that VE-465 causes the formation of abnormal prometaphase cells, affecting centrosome maturation and spindle bipolarity. These effects are entirely consistent with inhibition of Aurora A in the treated cells. VE-465 also induced mitotic abnormalities associated with Aurora B inhibition, namely dispersal of the chromosomes. It induced endoreduplication and cell cycle arrest as early as 24 h. At slightly higher concentrations, VE-465 induced apoptotic cell death in both Huh-7 and HepG2, as measured by annexin-V staining [80]. It has also been shown to have significant activity against paclitaxel-resistant ovarian carcinoma at higher doses, causing an 8-fold increase in apoptotic cell death at 100 nM [81]. Recently it was demonstrated to have significant activity against a panel of resistant and non-resistant multiple myeloma cell lines [82]. In this study, VE-465 inhibited proliferation of MM cells at concentrations of 400 nM or less. G2/M, 8n, and sub G1 populations were observed to accumulate with increasing exposure time, and correspondingly apoptotic markers appeared, including cleavage of PARP, caspase-3, 8, and 9. However, primary MM cells from patients are relatively insensitive to VE-465. Further it was shown that the effects of VE-465 were additive alongside with anti-MM agents [82]. In vivo efficacy was tested in Huh-7 xenograft model at 15, 25, and 35 mg/kg doses, administered twice daily for 14 days. These treatments caused reductions in the mean tumor volume of 59%, 59%, and 77% respectively [80]. In this study, VE-465 was also observed to inhibit histone H3 phosphorylation and to induce apoptosis in the tumors in a dose-dependent manner.

### JNJ-7706621

Johnson & Johnson’s JNJ-7706621 is an Aurora A and B kinase inhibitor. It was designed by the refinement of a series of acyl-substituted 1,2,4-triazole-3,5-diamine analogues [83]. The molecule has been reported to inhibit Aurora A and B with IC50 values of 0.011 and 0.015 μM, respectively [84]. However, in this study, it was also shown to inhibit CDK1 (cyclin dependent kinases), CDK2, CDK3, CDK4, and CDK6 (IC50 = 0.009 to 0.175 μM). Further, it inhibited proliferation of various cancer cell lines (IC50 = 0.112 to 0.514 μM), but showed less potency against normal cell lines, which were several-fold less sensitive. Moreover, it inhibited cell proliferation of both drug-sensitive and drug-resistant MES-SA cell lines at almost identical IC50 values, suggesting that PgP expression has no effect on JNJ-7706621 activity. Long-term effects of this compound on cell proliferation were determined by colony formation assay. HeLa cells were treated with either 1 μM or 3 μM concentrations for 48 h, followed by removal of the compound, and cells were then monitored for 7 days. Colony formation inhibition of 55% (1 μM) and 95.5% (3 μM) compared to control cells was reported. JNJ-7706621 induced apoptosis in the U937 histiocytic lymphoma cell line in a dose- and time-dependent manner, as evaluated by annexin-V staining. It also induced G2/M cell cycle arrest and polyploidy, which is one of the major phenotypic responses associated with Aurora kinase inhibition. The compound inhibited histone H3 phosphorylation at concentrations of 1 to 4 μM, which is again consistent with activity against Aurora B. The compound has been subjected to preclinical in vivo testing using the A375 human melanoma xenograft model, at doses of 100 and 125 mg/kg. Although daily dosing was the most efficient, five out of six test animals died after 22 days of treatment. However, an alternative ‘7 days on, 7 days off’ dosing schedule resulted in 93% TGI with no treatment-related deaths [84].

### CCT129202

Chroma’s CCT129202 has been shown to have high activity against Aurora A and Aurora B. It was developed through SAR optimization of an imidazopyridine scaffold. The compound has been reported to inhibit Aurora A, B, and C with IC50 values of 0.042, 0.198, and 0.027 nM, respectively [85]. It was also shown to cross-react with FGFR3, PDGFRβ (platelet-derived growth factor receptor), and GSK3β (glycogen synthase kinase 3 beta) at high concentrations. The effect of this compound on cancer cell line proliferation was tested on Colo205, SW620, HCT116, HT29, KW12, HeLa, A2780, OVCAR8, MDA-MB-157, and MV4-11 cells, and was found to have a half-maximal growth inhibition concentration (GI50) in the range 0.08 to 1.7 μM. In preclinical studies, it induced the accumulation of HCT116 cells with ≥4 N DNA content, accompanied by the appearance of subG1 apoptotic cells and accumulation of PARP cleavage. In HCT116 colon carcinoma cells, CCT129202 inhibited histone H3 phosphorylation after 15 min of treatment. The same effect was observed in the HCT116 xenograft model, i.e., inhibition of histone H3 phosphorylation after 15 minutes at a dose of 100 mg/kg. Furthermore, it induced stabilization of p53 (consistent with Aurora A inhibition). CCT129202 was administered at a dose of 100 mg/kg once a day for 9 days to HCT116 colon tumor xenografts in athymic mice to test its effects on TGI. The compound was well tolerated and induced significant TGI. Studies in mice also indicated that the compound has a favorable pharmacokinetic profile [85].

## AKIs in early preclinical studies

### Telik’s dual AKIs

Telik’s Aurora A and B inhibitors are at early preclinical stage. Telik’s Aurora inhibitors were designed by using proprietary drug discovery technology called TRAP (Target-Related Affinity Profiling). They have been reported to inhibit Aurora A, B, and VEGFR2 with IC50s of 1–10 nM [86]. Telik’s compounds have also been shown to inhibit proliferation of various colon, leukemia, lung, pancreatic, ovarian, and prostate cancer cell lines, with IC50s in the range 15 to 500 nM. Mechanistic actions consistent with Aurora inhibition were observed, including inhibition of histone H3 phosphorylation and polyploidy. The in vivo activity of TLK60404, one of Telik’s specific AKI was tested in human HCT116 and HL-60 mouse xenograft models. No toxicity or drug-related weight loss was observed. In addition, tumor growth was inhibited by 72% in HL-60 human xenograft model [86].

### AKI-001

Roche’s AKI-001 is an inhibitor of Aurora A and Aurora B that is in initial preclinical studies. AKI-001’s core, the pyridinyl pyrimidine amide scaffold, was discovered by high-throughput screening against Aurora-A kinase [87]. Further optimization and inclusion of lactam ring and hydrocarbon constraint to pentacyclic scaffold led to the discovery of the highly potent AKI-001 which is orally bioavailable phthalazine derivative with improved enzyme and cellular activity and a high level of kinase selectivity. AKI-001 has been shown to inhibit recombinant Aurora A and Aurora B at low nanomolar concentrations in ATP-competition assays. The compound also inhibited the proliferation of various cancer cell lines with IC50 values below 100 nM. In cellular assays, both Aurora A and Aurora B inhibition phenotypes were reported. AKI-001 had good oral bioavailability and was well tolerated at 5 mg/kg daily in the HCT116 xenograft model. At this dose AKI-001 induced 92% inhibition of tumor growth [87].

### CHR-3520

After screening many small molecule inhibitors, Chroma Therapeutics selected CHR-3520 for entry into preclinical studies. Initial studies have indicated that CHR-3520 is an inhibitor of AKs and other kinases related to cancer. Details of the specificity and cellular potency of CHR-3520 in relation to the AKs have not yet been disclosed [88].

### Other AKIs

In addition to these compounds, many biotechnology and pharmaceutical companies are developing novel AKIs. Cetek selected CTK110, an AKI with promising in vitro and in vivo anticancer activity, from a series of potential compounds. Ambit Biosciences have used their KinomeScan technology to select a lead AKI. KinomeScan is a novel and highly promising chemogenomics-based technique that is able to screen and characterize whole libraries of compounds across 400 kinases.

## First generation AKIs

### Hesperadin

Hesperadin is the first generation AKI discovered by Boehringer Ingelheim. Treatment of cancer cell lines with hesperadin resulted in Aurora B inhibition phenotype. The specificity of hesperadin towards Aurora A and C is unknown. Most of the basic functions of Aurora B in mitosis and its role in cancer cell proliferation were discovered by inhibiting it with Hesperadin [89].

### ZM447439

ZM447439, discovered by AstraZeneca, was the first AKI to be thoroughly characterized [90]. ZM447439 has been used extensively to study the biology of AKs and in their validation as targets for anti-cancer drug development.

## Natural AKI

### Jadomycin-B

Discovery of Jadomycin B (an Aurora B inhibitor) is attributed to structure-based virtual screening. Virtual screening against Aurora B (PDB code 2BFY) resulted in 22 compounds amongst a database of nearly 15,000 microbial natural products among which Jadomycin showed dose-dependent inhibition of Aurora B and several human cancer cell lines [25].

## Drug resistance to AKIs

Over the last 15 years, cancer chemotherapy has been greatly improved by the discovery of targeted drugs. In particular, some targeted drugs have achieved complete cures in some patients. However, the primary drug resistance or its development after few courses of chemotherapy is a major obstacle in the clinic. Many drug discovery companies are now focusing on drug resistance after realizing its importance in clinical rials and the clinic. Studies of drug-induced resistance in cell line models in parallel with preclinical development can be expected to yield significant information, and the findings of such studies can be used to circumvent drug resistance in clinical studies by designing combinations of anticancer agents.

Until recently, very little was known about drug-induced resistance mechanisms towards AKIs. One study found that SW620 (colon carcinoma) and MiaPaca (pancreatic carcinoma) cell lines became resistant to 1 μM AZD1152 over the course of three months exposure [91]. The resistant cells were maintained for further three months in the presence or absence of AZD1152. Genome-wide screening studies revealed that the expression of the ABCB1 (ATP-binding cassette, subfamily B, member 1/Multidrug resistance protein 1) gene was 70-fold higher in the SW620 AZD1152-resistant clones than in the SW620 cell line. At the same time, LC-MS (Liquid chromatography-mass spectrometry) analysis showed decreased drug accumulation in cytoplasm of resistant cells. When drug resistant SW620 cell line was treated with either 50 or 100 mg/kg of AZD1152, no decrease in tumor size was observed. By contrast, the MiaPaca pancreatic carcinoma cell line became resistant to AZD1152 following overexpression of the ABCG2 (ATP-binding cassette, subfamily G, member 2/Breast cancer resistance protein) drug transporter. Microarray analysis revealed that the expression of this gene was increased 98-fold relative to controls. [91].

Seamon et al. obtained JNJ-7706621 resistant HeLa cell line by exposing the cells to increasing concentrations over a 12 month period [92]. A LC-MS study on these resistant lines showed a highly significant reduction of intracellular drug accumulation. Quantitative RT-PCR (Real time polymerase chain reaction) studies revealed a 163-fold increase in ABCG2 (BCRP/Breast cancer resistance protein) transporter gene expression, a 37-fold increase in ABCC2 (ATP-binding cassette, subfamily C, member 2), and a 3-fold increase in ABCB1. Treatment of the resistant HeLa cell line with the ABCG2 inhibitor fumitremorgin C restored the sensitivity to JNJ-7706621 and mitoxantrone [92].

Unlike previous studies, in which resistant cell cultures were developed by prolonged exposure to slowly-increasing levels of AKIs, Girdler et al. [93] treated the HCT116 cell line with a supralethal dose of ZM447439 (1 μM) for four weeks. While most of the cells died, 20 drug resistant colonies were appeared and among them seven clones were selected for further characterization. Colony formation and cell proliferation assays demonstrated that the clones R1 and R2 were highly resistant to ZM447439 compared to controls. cDNA sequencing of Aurora B from these resistant clones (designated R1-R7), revealed five point mutations. R3, R4, and R6 harbored two point mutations. H250Y was common to all three of these clones, whereas G160V was specific to R3 and R4, while G160E was specific to R6. The R1, R2, R5, and R7 clones contained the G160E, Y156H and L308P, H250Y, and Y156H mutations, respectively. Ectopic expression of the Y156H, G160V, and H250Y mutants in DLD-1 cells revealed that they retained catalytic activity. The Y156H genotype along with G160V and G160E showed strong cross-resistance to VX-680 and hesperadin, but not H250Y. Advanced crystallographic studies revealed that these Aurora B mutations increase the steric hindrance in the active site of Aurora B, inhibiting the binding of ZM447439, but not that of ATP [93].

## Validity of Aurora kinases as oncology targets

Although AKs are widely considered as oncogenes, many questions were raised regarding their role in cancer initiation. Despite their overexpression in many tumors, no clear role for the AKs in tumorigenesis has been established. Probably the overexpression of AKs may not be the main cause of cancer initiation in primary tumors, rather it could be a late event. Bischoff et al*.* showed that overexpression of wild type Aurora A is sufficient to transform rat1 and NIH3T3 fibroblasts. Authors also speculated that additional oncogenic events may be required for transformation [94]. However, another similar study performed by Tatsuka et al., did not observe transforming potential of Aurora A alone in BALB/c 3T3A31-1 cells. Interestingly in co-transfection study, Aurora A forced expression potentiated G12V H-Ras induced transformation [95]. Ota et al*.* reported that overexpression of Aurora B induced histone H3 phosphorylation (Ser10) and mitotic phenotype in Chinese hamster embryo cells [96]. Further, when these cells were xenografted into mice, they were able to form aggressive and invasive tumors compared to control cells that express low Aurora B. Nonetheless, another similar study performed by Kanda et al*.* did not observed transforming potential of Aurora B alone in BALB/c 3T3 A31-1-1 cells. Here also forced expression of Aurora B augmented the frequency of G12V H-Ras induced transformation [97]. Role of Aurora C in transformation has not been yet established. All these studies clearly suggest that AKs may not be directly involved in cancer initiation, but rather cooperate with or complement other oncogenes. Overexpression of AKs and their association with poor prognosis were reported consistently in many cancers, indicating that AKs are required for tumor maintenance, progression, and survival. These important functions of AKs are sufficient to consider them as viable targets in cancer disease, even though their clinical validation is still awaited.

Among the AKs, which Aurora kinase is the best target for effective cancer treatment has become an interesting topic of debate. Some reports suggest Aurora A inhibition has more cytotoxic than cytostatic effects [98], while others suggest targeting Aurora B is more effective [99]. MLN8237 is a highly specific and potent Aurora A inhibitor, which has been shown to induce apoptosis rapidly compared to other AKIs. It has also been shown to have anticancer activity on a wide range of cancer cell lines, such as MM cell lines. In clinical studies, it produced few side effects and had good pharmacokinetics and efficacy. Interestingly, MLN8237 has also displayed higher anticancer activity than standard agents in childhood cancer cell lines and their xenograft models. However, its efficacy under the pediatric clinical setting needs further studies. AZD1152 is a specific and potent Aurora B inhibitor, which is currently at the forefront of clinical studies compared to other Aurora inhibitors. It has been found to induce anticancer activity in both leukemias and solid tumors. It also induces rapid apoptosis in many cancer cell lines, suggesting it has cytotoxic activity. After AZD1152 administration, 15 patients with progressive cancer showed stable disease.

## Dose limiting target toxicities of AKIs

Under in vitro conditions AKIs displayed broad anticancer activity in rapidly proliferating cancer cells, but not in resting cells. Hence it is likely that chemotherapy with AKIs may be toxic to rapidly dividing hematological cells. As anticipated, the most common on-target toxicity reported for many AKIs is grade 3/4 neutropenia. The other on-target toxicities reported include wide range of hematological toxicities including leukopenia and myelosuppression. Few cases of septicemia and pneumonia were also reported and they may be the consequences of neutropenia. On the other hand off-target toxicities were also reported for AKIs, which includes hypertension, somnolence, mucositis, stomatitis, proctalgia, grade 3 increase in aspartate aminotransferase, and grade 2 ventricular dysfunction. Importantly most of the side effects were reversible upon drug withdrawal. VX-680 caused cardio-toxicity and was associated with death of one patient, which prompted to suspend the compound from clinical trials recently. Probably the off-target toxicities of AKIs could be due to their cross-reaction with other kinases, since their spectrum varies from among individual AKIs.

AKIs described in this review displayed much high potency in hematological cancers both under in vitro and in vivo conditions compared to solid tumors. This clearly suggests that AKIs are highly active in rapidly cycling cells. This point corresponds to the dose limiting toxicity including bone marrow suppression and associated neutropenia in normal hematological cells. Drug related toxicities of AKIs on hematological cells and associated bone marrow toxicity was reported exceptionally well by Wilkinson et al*.* [71]. Bone marrow tissue from the AZD1152 treated rats were used to study the effect on rapidly dividing cells. Staining of the tissue revealed signs of atrophy associated with decrease in the total cellular content. However, myelosupression was reversed within a week of AZD1152 withdrawal. Clinicians should consider intermittent dosings at appropriate intervals or metronomic therapy in order to better target tumor cells and allowing bone marrow cells to recover.

Administration of hematopoietic growth factors in conjunction with anticancer drugs may also help to reduce the severity of bone marrow toxicities. Many clinical studies were performed using growth factors in conjunction with anticancer agents and were successful to limit the bone marrow related toxicities [100]. In the context of AKIs, Cohen et al*.* used G-CSF in conjunction with PHA-739358 [34]. In this study they were able to escalate the PHA-739358 dose until 1000 mg/m^2^ and did not reported any bone marrow related toxicities, particularly neutropenia. This was the first time they were able to achieve objective responses in patients with advanced solid tumors. On the other hand, grade 3/4 neutropenia and neutropenic infection were reported in the absence of G-CSF at around 500 mg/m^2^ dose. Conjunctive use of growth factors would be beneficial in significantly reducing drug associated toxicities and also to enhance the efficacy of drugs by dose intensification.

## Additional features of AKIs

From our review, one can appreciate many important aspects of AKIs. Firstly, almost all the AKIs cross-reacts with many structurally related oncogenic kinases including VEGFR2, FLT3, Bcr-Abl, JAK, and FGFR1. It has been consistently reported that these kinases are involved in the initiation and its progression of cancer. The main advantage of targeting multiple kinases other than AKs is that majority of cancers have abnormalities at multiple targets, thereby increasing the probability of effective treatment. Targeting multiple kinases may also prevent the emergence of resistance during AKI therapy, because resistance towards AKIs in the clinic is highly probable. To support this, recently Girdler et al*.* reported emergence of resistance in HCT116 cell line due to Aurora B mutations [93].

Secondly, we noticed that some pan-AKIs are significantly effective in drug resistant cancer lines. They were able to inhibit the cell proliferation equally or more efficiently compared to non-resistant parent cell lines. The authors of these studies were also able to successfully validate the AKIs ability to overcome the (multidrug) resistance in xenograft models at least with few AKIs. Hence AKIs may have a huge potential to overcome the resistance in patients with refractory cancers and some clinical studies are underway. Imatinib targeted towards Bcr-Abl has been highly successful in treating CML patients with Bcr-Abl translocation. In some patients complete hematological and cytological responses were achieved. However, during the course of imatinib chemotherapy, many patients acquired Abl kinase domain mutations resulting in imatinib resistance. Among multiple mutations, the gate keeper mutation, namely T315I is very aggressive, which renders complete resistance to imatinib and related compounds. Many AKIs were able to inhibit T315I Bcr-Abl mutations with high specificity than wild type Bcr-Abl. AKIs have also been proved to be efficient in reversal of resistance in T315I Bcr-Abl CML cell lines both in vitro and in vivo. This sparked the interest of testing the AKIs in refractory CML patients with T315I mutations and many clinical studies are underway. Discovery of AKIs efficiency in inhibiting T315I Bcr-Abl formed a strong rationale in testing the existing second generation kinase inhibitors on drug resistant mutants.

Thirdly, AKIs have a great potential to enhance the efficacy of other anticancer drug and radiation therapies, which can be exemplified by some reports. MK-5108 significantly enhanced the efficacy of docetaxel in HeLa-S3 and ES-2 cell lines both in vitro and in vivo [67]. Low concentrations of VE-465 alone synergized with paclitaxel and induced 4.5 fold greater apoptosis in 1A9 ovarian cancer cell line [81]. On the other hand AZD1152 [101] and PHA-680632 [79] greatly enhanced the effect of radiation treatment. These findings have potential interest for further clinical development. AKIs have broad anticancer activity in most of the cancer cell lines tested. The above described characteristics of AKIs make them very attractive candidates for targeted therapy.

## Potential approvable AKIs for routine clinical use

In preliminary clinical studies, AKIs have consistently displayed cytostatic effects, tumor response or stable disease, particularly in solid tumors. However, because of the plethora of synthetic AKIs with diverse chemical structures, target and off-target activities, toxicological profiles, and efficacy, it is difficult to predict which compound(s) will enter clinical use. Certainly, one of the most interesting and advanced inhibitors is PHA-739358. This compound not only inhibits AKs, but it also has an off-target effect on Abl, Ret, and FGFR-1 oncogenic kinases, which are implicated in many types of malignancies. Moreover, it has been shown to have good pharmacokinetic properties combined with high anticancer activity; 28/80 patients with solid tumors showed stable disease, which lasted for 6 months in six patients. As a result of these attributes PHA-739358 is regarded as a highly promising clinical candidate. Metastasis of a malignant tumor is one of the hallmarks of cancer and its progression. Hence, inhibition of metastasis by suppressing angiogenesis is a novel approach for cancer treatment. Interestingly, CYC116 and ENMD-2076 inhibits VEGFR-2, which directly promotes angiogenesis. These drugs have also exhibited significant anticancer effects on a broad range of cancer cell lines. These properties strongly encourage their further clinical development, which could improve overall survival of patients. AT9283 is a promising multikinase inhibitor with activity against, e.g., AKs, JAK2, JAK3, and Abl. In one clinical trial, 30% of patients showed stable disease, and the compound was well tolerated. One of the common mechanism of cancer cell drug resistance is the overexpression of PgP, which actively effluxes the anticancer agent before reaching the target. Overexpression of PgP and associated multidrug resistance was reported in cancer patients that are resistant to many anticancer agents. AMG-900 was able to overcome the resistance particularly in PgP upregulated multidrug resistant cancer cell lines. AMG-900 may have great potential in both enhancing the therapeutic potential of anticancer agents and also in combating drug specific resistance.

## Clinical efficacy of Aurora kinase inhibitors

Though AKIs have showed broad range anticancer activity in cell lines and xenograft models, they did not lived up to the expectations in the clinic. Here we speculate some of the reasons; firstly the rationale for targeting AKs is not validated. The main reason for targeting AKs is based on the fact that they are upregulated in many cancer types. It could be possible that tumors cells may not be addicted to AKs for their proliferation and survival as much as to Bcr-Abl, K-ras, or B-Raf oncogenes. Recently many questions were raised regarding the validity of AKs as oncogenes. Probably the role of AKs in inducing malignant phenotype is transient. This is probably one of the reasons that AKIs were not specifically efficient in any cancer type, compared to other routinely used anticancer agents in the clinic. For example platinum drugs are well known agents to treat ovarian and breast cancers, paclitaxel for ovarian and breast cancers, gemcitabine for pancreatic and lung cancers, and bortezomib and thalidomide for multiple myeloma. Secondly, polo-like kinases (PLK) share similar functions that are assigned to AKs [101]. This is strongly supported by the fact that cellular phenotypes overlap with the inhibition of PLKs and AKs. Thus it is likely that PLKs may complement the functions of AKs and may compromise the AKIs induced effects. Hence outcome of combined inhibition of AKs and PLKs might be desirable compared to single target inhibition. Thirdly, mitotic inhibitors are well validated to treat cancer cells in vitro and in xenograft models, where tumors doubling times are relatively short. However, in real life situation human tumor cells have very long doubling times that may range from months to years. AKIs execute their mode of actions only when the cells are actively proliferating, as AKs are predominantly expressed in mitotic phase. In clinical trials, AKIs mostly induced only stable diseases (only in few patients), but rarely the partial or complete responses and this could be due to high doubling time of tumors cells. Most of the AKIs doses employed in the Phase I studies are relatively low, ranging from 3 to 200 mg/kg, due to DLTs, mainly neutropenia. These doses are well below the therapuetic window for activity. Thus further dose enhacements in Phase II studies in conjunction with growth factors would be beneficial. Cohen et al*.* succesfully escalated the doses upto 1000 mg/m^2^ in the presence of G-CSF and reported objective responses with reduced toxicities [34]. One of the solutions could be intermittent dosings at appropraite intervals or metronomic therapy to recover bone marrow cells should be considered.

## Conclusions

The AKs have been the focus of considerable attention since their discovery in *Drosophila* mutants, and many independent studies have contributed to our understanding of their biology. AKs have multiple important functions in mitosis, and their overexpression in some cancers prompted the discovery and development of novel AKIs as therapeutic drugs using a variety of experimental and computational techniques. Less than ten years after AKs were discovered in humans, more than ten Aurora inhibitors had entered clinical trial, and the number of new AKIs entering preclinical development or clinical trials is continuing to increase. No general mechanisms of tumor cell resistance to AKIs have yet been identified, although some preliminary studies suggest that mutations of the targeted Aurora kinase and overexpression of drug-resistance genes may be involved.

Further in-depth clinical studies are now required to evaluate the effectiveness of AKIs. Hence, it is too early to draw any conclusions regarding which compounds are likely to enter the market for routine use. Furthermore, identification of biomarkers based on gene expression studies, that are predictive of anticancer activity for a specific drug in individual patients is important. Some AKIs have been shown to be very effective in single agent or combination studies in some patients. Widely accepted functional pharmacological/surrogate biomarkers are available for both Aurora A and B inhibition, which makes them attractive targets. In the absence of tumor associated biomarkers, neutropenia per se is a biomarker of Aurora kinase inhibition in the bone marrow cells. Thus, biomarkers that allow the efficacy of given AKI to be assessed offer the promise of individualized therapy, which academic clinicians are keen to pursue. Since AKIs are emerging as targeted cancer therapeutics with interesting off-target effects, one might reasonably hope that they could also be used to tackle the problem of resistance, and thus enhance the treatment of cancer.
